# Medication Adherence Among Diabetic Patients in Madinah, Saudi Arabia: Interplay of Cultural Beliefs, Socioeconomic Status, and Clinical Determinants

**DOI:** 10.3390/jcm14196717

**Published:** 2025-09-23

**Authors:** Muayad Albadrani, Asrar Alharbi, Shahad Aljohani, Reenad Al Harbi, Taif Alluhaybi, Esraa Alammash, Afrah Aljabri, Naweed SyedKhaleel Alzaman

**Affiliations:** 1 Department of Family and Community Medicine and Medical Education, College of Medicine, Taibah University, Madinah 42353, Saudi Arabia; 2 Health and Life Research Center, Taibah University, Madinah 42353, Saudi Arabia; 3 College of Medicine, Taibah University, Madinah 42353, Saudi Arabia; 4 Department of Medicine, College of Medicine, Taibah University, Madinah 42353, Saudi Arabia

**Keywords:** diabetes, medication adherence, cultural beliefs, socioeconomic factors, cross-sectional, Saudi Arabia

## Abstract

**Background/Objectives**: Chronic diseases, such as diabetes mellitus, require sustained management and medication adherence to reduce the risk of related complications and mortality. However, the adherence levels are not satisfactory, which could be attributed to several factors, including cultural beliefs and socioeconomic factors. This study aimed to assess the relationship between cultural and socioeconomic factors, patient preferences, and medication adherence among diabetic patients. **Methods**: A mixed-methods cross-sectional design was implemented using face-to-face questionnaires and personal interviews. This study was conducted in 159 primary healthcare clinics (PHCs) in Madinah, Saudi Arabia, from 26 August 2024 to 10 February 2025. It included type 1 and type 2 diabetic patients. The Morisky Medication Adherence and General Medication Adherence Scales were used to evaluate diabetes medication adherence among the participants. **Results:** The included 424 diabetic patients had a predominant age range from 40 to 59 (48.1%). The majority were non-smokers (88.7%), Saudi Arabian (94.6%), and female (62.7%). The findings revealed a significant association between patient age (*p* < 0.001), body weight (*p* = 0.023), nationality (*p* = 0.015), educational level (*p* = 0.027), and the presence of comorbidities (*p* = 0.005) with the level of medication adherence. **Conclusions**: This study revealed that most diabetic patients attending PHCs in Madinah exhibited medium-to-high levels of medication adherence, with key influencing factors including age, comorbidities, education level, physician satisfaction, and health self-awareness.

## 1. Introduction

Diabetes mellitus is a chronic metabolic disease causing global health concern, as its incidence is continuously rising, specifically that of type 2 diabetes, which accounts for 90% of diabetes cases [1]. According to global, regional, and national diabetes prevalence estimates for 2024 and projections for 2050, published by the International Diabetes Federation’s Diabetes Atlas, 589 million adults (11.1%) were living with diabetes in 2024, and this number is projected to rise to 853 million by 2050 [2]. Additionally, the prevalence of diabetes will increase by 10.9% in 2045, with the number of cases possibly reaching 700 million [3]. In Saudi Arabia, the last ten years have seen a notable increase in diabetes cases of 8%, which is alarming because, at present, 25% of the Saudi population has diabetes [4]. Consequently, prevention and treatment strategies, including anti-diabetic therapies and lifestyle changes, are mandatory to control diabetes and lower the incidence of prediabetes [5]. In particular, adherence to insulin or oral medications has been proven to provide better health outcomes and patient satisfaction. Additionally, research has found that diabetes medication adherence reduces the risk of related complications, such as nephropathy, retinopathy, and foot complications, as well as hospitalization and mortality rates [6].

However, the current literature has revealed that one in every three diabetic patients does not adhere to the prescribed medication regimen [7]. Moreover, each 10% reduction in adherence is linked to a 0.14% increase in glycosylated hemoglobin (HbA1c), leading to serious complications and higher rates of emergency visits [8]. According to the World Health Organization (WHO), half of the population adheres to chronic disease medications in developed countries and less than half in underdeveloped countries. This results in higher mortality rates, thereby placing a financial burden on the patient and the healthcare system [9]. Although there is no standardized method for assessing medication adherence, the Morisky Medication Adherence Scale (MMAS) is a validated, reliable, and self-reported tool used for this purpose, and it could be beneficial for detecting the potential factors affecting such adherence [10].

Several studies have reported some of the cultural and socioeconomic factors that influence the medication adherence of diabetic patients. Researchers have observed that social support and self-efficacy play key roles in adherence [11,12]. Furthermore, financial and employment status have a strong association with the degree of adherence [13]. In addition, trust between the patient and the physician has a substantial effect on medication adherence, which could be achieved by proper communication and a better understanding of medication [14].

As medication adherence is a prerequisite for better health outcomes, besides lifestyle changes, a comprehensive understanding of the cultural beliefs and socioeconomic factors that could impact this adherence is important. This could benefit primary healthcare professionals in improving their quality of care and avoiding serious complications. Thus, this study aimed to assess the relationship between cultural and socioeconomic factors, patient preferences, and medication adherence among diabetic patients.

## 2. Methods

### 2.1. Study Design and Setting

This was a cross-sectional observational study that used face-to-face questionnaires and personal interviews. This study was conducted in 159 primary healthcare clinics (PHCs) in Madinah, Saudi Arabia, from 26 August 2024 to 10 February 2025.

### 2.2. Study Population

Data were collected from diabetic patients attending PHCs in Madinah City, Saudi Arabia.

#### 2.2.1. Inclusion Criteria

A diverse group of patients with type 1 and type 2 diabetes was included, ensuring representation of various cultural and socioeconomic backgrounds.

#### 2.2.2. Exclusion Criteria

The exclusion criteria were patients without diabetes, those with mental health or psychiatric conditions, and those who did not understand either English or Arabic.

#### 2.2.3. Sampling Technique and Sample Size

A convenient sampling technique was used. The sample size was calculated using the Raosoft online sample size calculator. Considering a margin of error of 5%, a confidence level of 95%, and maximum uncertainty, a minimum of 377 participants was required, and this was rounded up to 400 to account for approximately 10% invalid or incomplete responses.

### 2.3. Data Collection Method and Tools

Data were collected using face-to-face questionnaires and personal interviews. The questionnaire included sociodemographic characteristics (gender, age, weight, height, smoking status, marital status, educational level, and health insurance coverage) and dependent variables, such as the patient’s beliefs towards diabetes mellitus, preferences regarding treatment modalities, dietary adjustments, lifestyle changes, confidence in disease management, understanding of medications, medication adherence behaviors, and challenges faced. Age was stratified into three groups (<40, 40–59, and ≥60 years) to reflect younger, middle-aged, and older adult life stages commonly used in epidemiological studies and to ensure adequate sample sizes for subgroup analyses. The Morisky Medication Adherence Scale-4 (MMAS-4), specifically adapted for diabetes medication adherence, was utilized. This scale comprises four items that assess various aspects of medication-taking behavior, including forgetting, carelessness, stopping medication when feeling better, and stopping medication when feeling worse. The term “modified” refers to its specific application and interpretation in the context of diabetes management, drawing on established factors relevant to adherence in this patient population, as identified in previous research. Participants were divided into two groups based on their adherence scores: 4 (high adherence) and 3 to 0 (medium or low adherence). In addition to the MMAS-4, the General Medication Adherence Scale (GMAS) was also employed; the use of both scales stemmed from their complementary strengths in assessing medication adherence. The MMAS-4, a widely validated and concise tool, allows for the quick screening of non-adherence behaviors, particularly identifying intentional and unintentional barriers. Conversely, the GMAS offers a more comprehensive and nuanced assessment of adherence across various behaviors, utilizing an 11-item Likert scale to capture a broader spectrum of adherence levels (e.g., high, good, partial, low, and poor). This dual approach allowed for a robust evaluation of adherence, with the MMAS-4 providing a rapid screening approach and the GMAS providing a more detailed, multi-faceted understanding, thereby enhancing the validity and reliability of our adherence measures. Following the administration of the quantitative questionnaire, a semi-structured qualitative interview was conducted with a subset of patients. These interviews aimed to gain deeper insights into the participants’ beliefs, perceptions, and experiences related to diabetes self-management, treatment adherence challenges, and facilitators. Key areas explored included personal understanding of their condition, barriers to healthy lifestyle choices, motivations for adherence, and interactions with healthcare providers. During these interviews, recent HbA1c values were extracted from participants’ electronic laboratory records for tests conducted within the three months prior to questionnaire administration to assess diabetes control. The qualitative interview data were collected and will be analyzed and reported in a separate, dedicated qualitative study to allow for a comprehensive exploration of the rich thematic insights into patient experiences.

### 2.4. Statistical Analysis

IBM Statistical Package for the Social Sciences (SPSS) software, version 26.0, was used for data analyses. A descriptive analysis was performed to describe categorical data using numbers and proportions. For numerical data, the median and interquartile range (IQR) were used for non-normally distributed data after conducting the Shapiro–Wilk test. Associations between categorical variables were assessed using Pearson’s chi-square test. The Mann–Whitney and Kruskal–Wallis tests were used to assess the relationship between cultural and socioeconomic factors and the participants’ adherence to medication. A *p* < 0.05 was used to determine statistical significance.

The Morisky Medication Adherence Scale (MMAS-4) was utilized to assess the level of adherence to diabetes medication. Respondents who answer “no”, indicating the patient did not exhibit the non-adherent behavior, receive a score of 1, while those who answer “yes” receive a score of 0. Data have been coded using validated cutoffs (High = 4, Medium = 2–3, Low = 0–1). The General Medication Adherence Scale (GMAS) is a self-reporting tool consisting of 11 items. Each item is scored using a 4-point Likert scale: “Always” scores 0, “Mostly” scores 1, “Sometimes” scores 2, and “Never” scores 3. Respondents receive a score based on their adherence level, with a maximum possible score of 33. To obtain the final score, the scores of all items are added together, allowing for an assessment of adherence categorized as follows: high (30–33), good (27–29), partial (17–26), low (11–16), or poor (10 or below).

### 2.5. Ethical Considerations

The Institutional Review Board (IRB) of the General Directorate of Health Affairs in Madinah granted approval for this research under ethical ID 24-078 on 18 August 2024. The committee is registered with the National Registration Number NCBE-KACST, KSA (H-03-M-84). Permission to use the MMAS-4 was granted by its developer, Dr. Donald E. Morisky. This scale is copyrighted (U.S. Reg. No. TX-8-285-390) and is licensed under Certificate Number 0942-6773-4869-8100-6082, issued on 20 May 2025. Proper attribution and usage were adhered to according to the terms outlined at www.adherence.cc (accessed on 25 July 2025). All procedures followed relevant guidelines and regulations, including the Declaration of Helsinki. Written informed consent was obtained from the participants. All information provided by the study participants was kept confidential and anonymous.

## 3. Results

This study included 424 diabetic patients from PHCs in Madinah City, Saudi Arabia. The participants were mostly middle-aged (40–59 years), Saudi Arabian, and female, and the majority had a university education, were unemployed, and lacked health insurance coverage. Most participants were non-smokers and reported receiving help with their diabetes care. All details are presented in Table 1.

Regarding clinical and medication profiles, type 2 diabetes was predominant among the participants. More than half of the patients did not have diabetes-related complications, but the majority had uncontrolled diabetes, with HbA1c above 6.5%. Most patients primarily used oral diabetes medications, did not rely on herbal medications, and reported receiving diabetes education and regular doctor visits. All clinical details are available in Table 2.

The patients’ beliefs towards diabetes mellitus varied, with a high proportion disagreeing that diabetes only occurs with high blood sugar levels, has low consequences, or has minimal symptoms. Conversely, the majority agreed that diabetes interferes with their social life, that diabetes medication could lead to addiction, and that they had low control over their diabetes. Table 3 provides a comprehensive overview of the participants’ beliefs.

**Figure 1:**
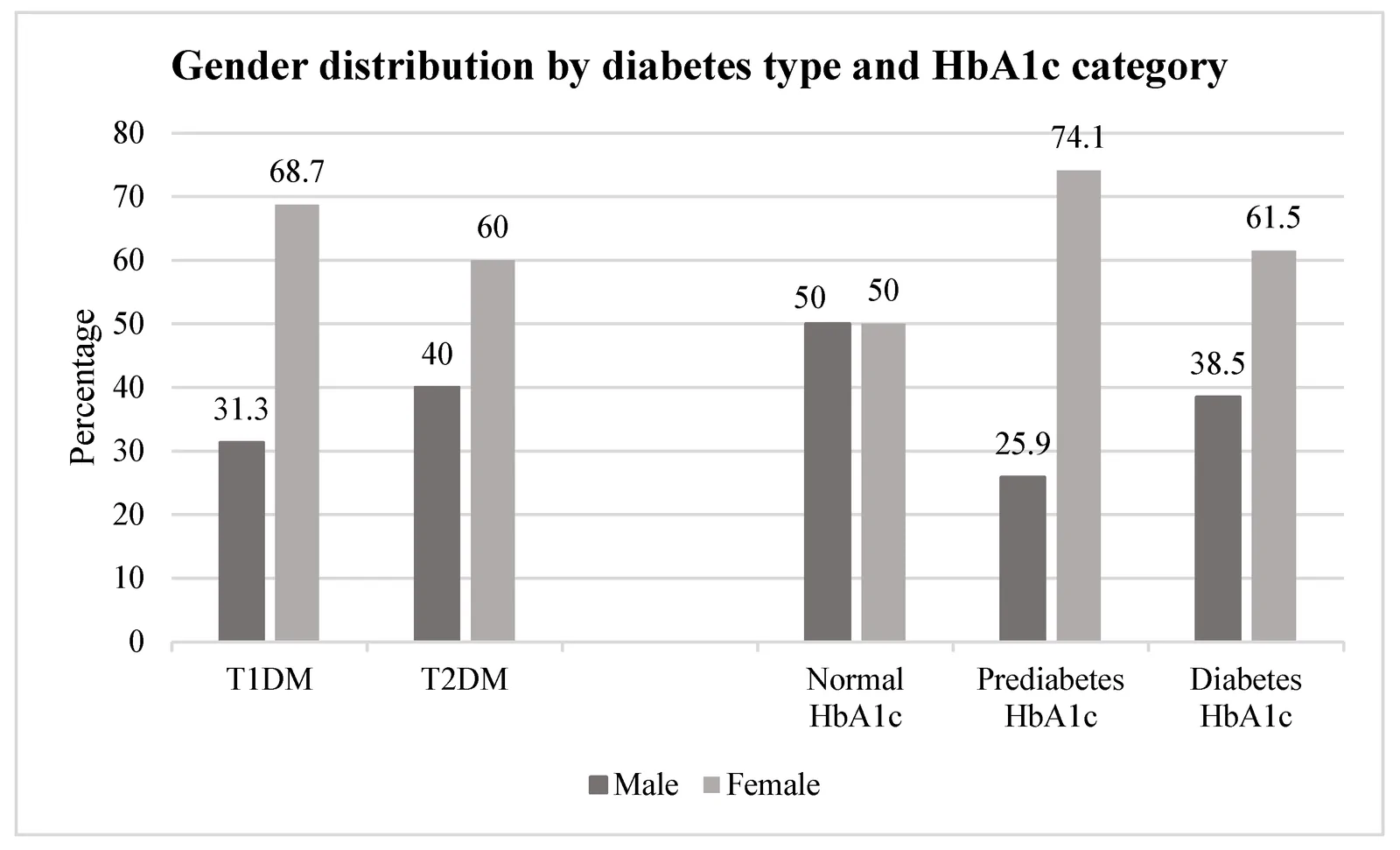
Gender distribution by diabetes type and HbA1c category (N = 424). HbA1c categories: normal < 5.7%; prediabetes 5.7–6.4%; diabetes ≥ 6.5%.

Figure 1 illustrates the gender distribution across diabetes type and HbA1c categories. Among participants with type 1 diabetes, 31.3% were male, and 68.7% were female; for type 2 diabetes, 40.0% were male, and 60.0% were female. Normal HbA1c was 50.0% in males versus 50.0% in females, prediabetes HbA1c was 25.9% in males versus 74.1% in females, and diabetes HbA1c was 38.5% in males versus 61.5% in females. Chi-square tests showed no significant differences by gender for diabetes type (*p* = 0.087) or HbA1c category (*p* = 0.124).

**Figure 2:**
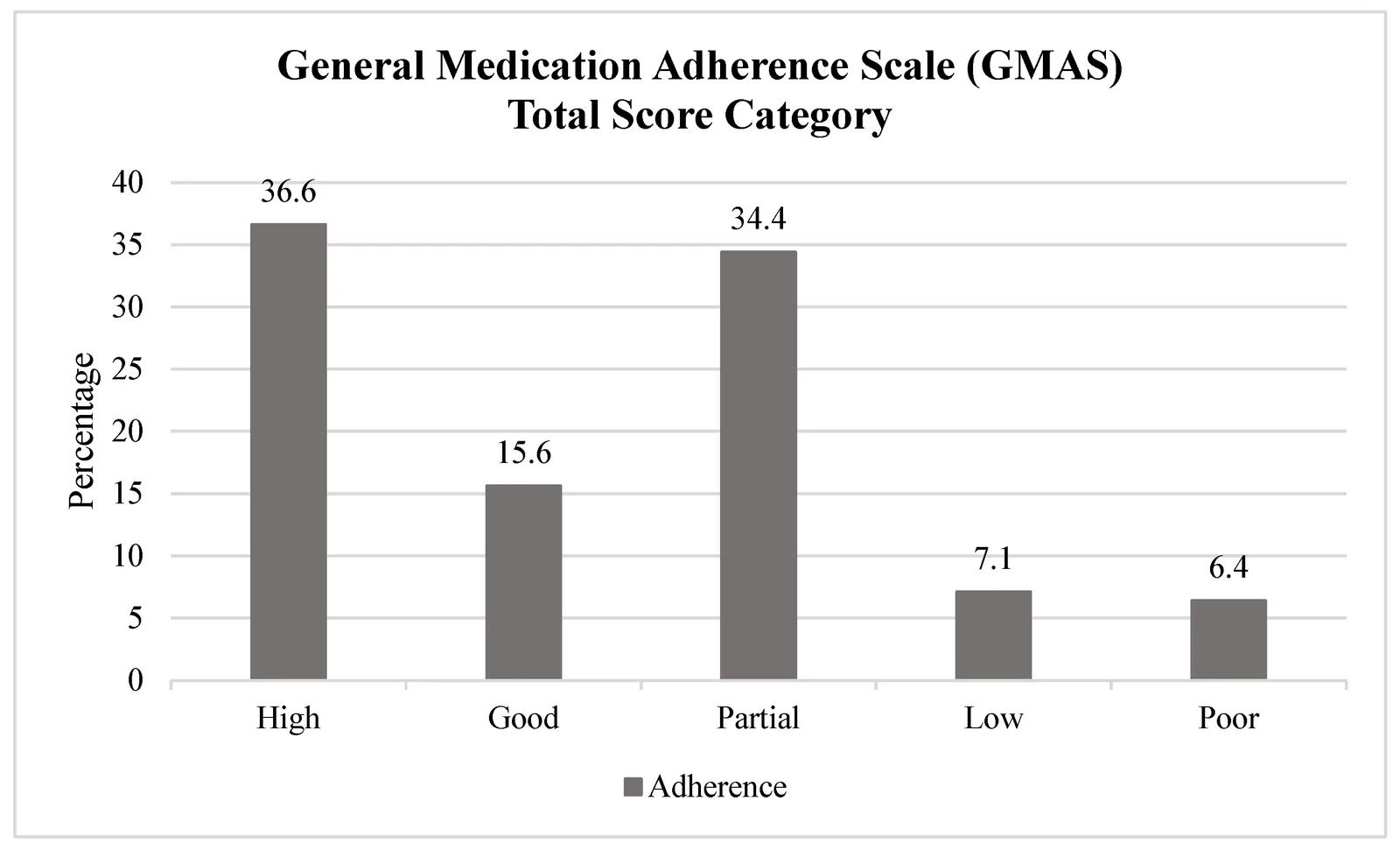
General Medication Adherence Scale (GMAS)—total score category (N = 424). GMAS categories: high (30–33), good (27–29), partial (17–26), low (11–16), or poor (10 or below).

**Figure 3:**
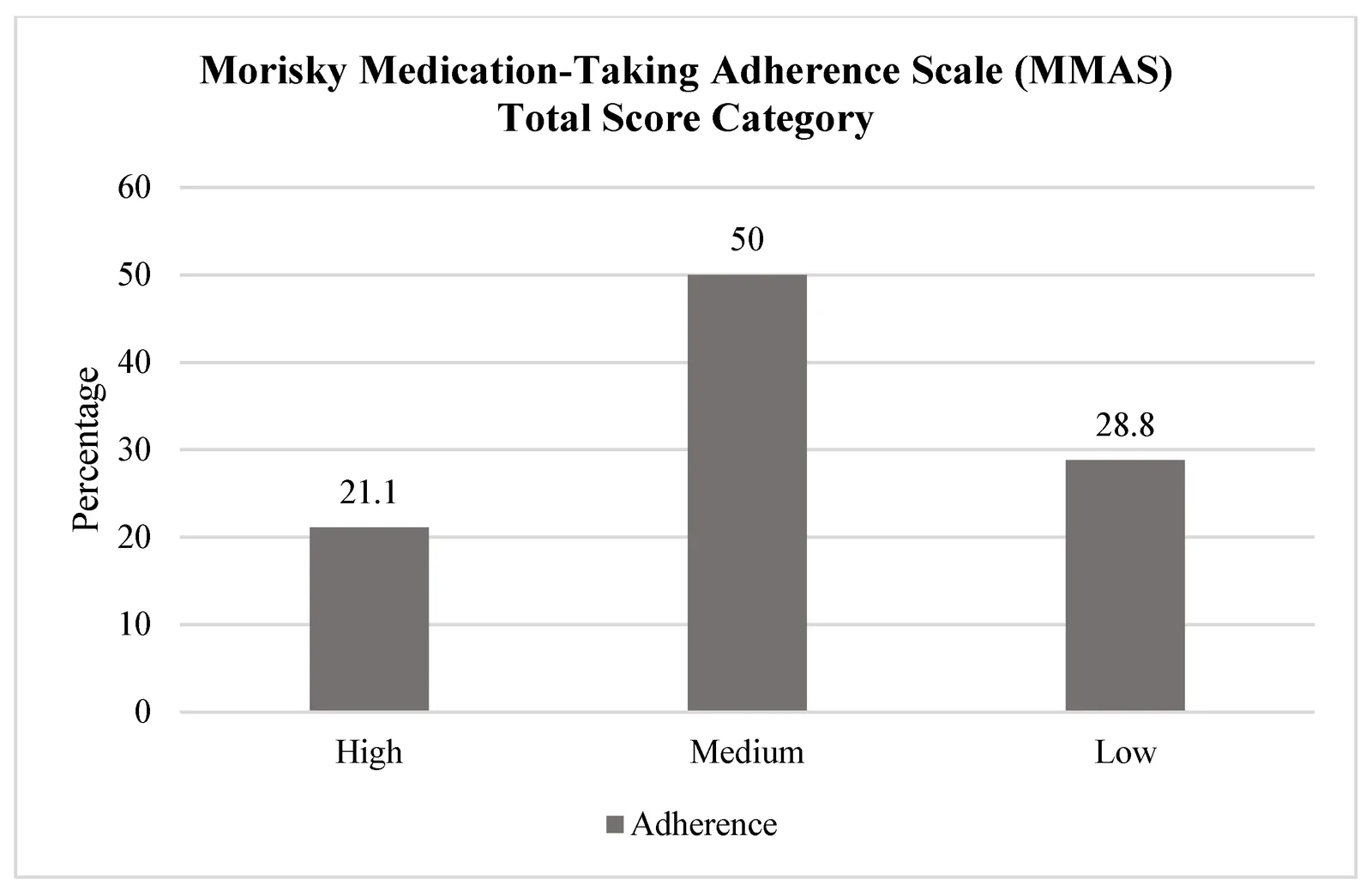
Morisky Medication-Taking Adherence Scale (MMAS)—total score category (N = 424). MMAS categories: high (4), medium (2–3), or low (0–1).

Regarding medication adherence levels, Figure 2 illustrates the distribution of adherence categories based on the GMAS. A notable proportion of the participants, specifically 36.6%, demonstrated high medication adherence according to the GMAS. This suggests a moderate level of overall adherence within the study population. Similarly, Figure 3 presents the medication adherence distribution, as measured using the MMAS, revealing that half of the diabetic patients (50%) exhibited medium adherence.

A significant association was found between higher adherence and older age (*p* < 0.001; *p* = 0.004), being overweight (*p* = 0.023), Saudi nationality (*p* = 0.015), and holding a higher education level (*p* = 0.027). Additionally, the presence of comorbidities (*p* = 0.005), taking five or more medications (*p* < 0.001), having type 2 diabetes (*p* = 0.024), and a diabetes duration of more than 10 years (*p* = 0.002) were associated with higher adherence levels. More significant results are presented in Table 4.

Regarding beliefs, adherence was significantly higher among those who were satisfied with their physician (*p* = 0.008) and those who disagreed with negative beliefs (e.g., fear of addiction and side effects, low diabetes control and confidence in management, lack of symptoms, and not needing medications with normal blood sugar) (*p* < 0.001). The patients who disagreed with specific misconceptions about diabetes (e.g., diabetes occurring only with high blood sugar levels or having low consequences) showed significantly higher adherence on the GMAS (*p* = 0.022 and *p* = 0.01, respectively). All results are presented in Table 5.

## 4. Discussion

Patient adherence to medications prescribed for chronic illnesses such as diabetes mellitus is an essential factor for successfully achieving good glycemic control and proper management of the disease. However, some patients find it difficult to adhere to their medication, especially if its duration is lifelong. Research has shown that about 59.8% of type 2 diabetic patients have poor medication adherence [15], which can be attributed to various influencing factors. This study aimed to evaluate the relationship between cultural and socioeconomic factors, patient preferences, and medication adherence among diabetic patients.

This study revealed that medication adherence among diabetic patients in Madinah was generally moderate. Notably, we found that older age, higher education, and a longer diabetes duration were positively associated with higher adherence. This aligns with the observations of Shaha et al. [16], who also noted that elderly patients with higher secondary education and those with more than 10 years of diabetes were more adherent. While Shaha et al. also reported higher compliance among urban and employed patients, we did not observe significance regarding employment status or residential area, likely because almost half of our population was unemployed, and most resided in urban areas. These results indicate that accumulated self-management skills and improved health literacy, often gained through sustained experience with the disease, contribute to better adherence over time.

Interestingly, patients with comorbidities and those prescribed multiple medications both demonstrated higher adherence, suggesting heightened disease awareness, increased motivation to manage health, and enhanced engagement with healthcare providers that fosters routine medication-taking behaviors. These integrated findings align with a Tanzanian study [17] and Gast et al. [13], despite contrasting results reported by Al-Noumani et al. [14], and underscore the positive influence of self-efficacy, provider support, and confidence in disease management on adherence in chronic conditions [18,19,20].

A notable finding was the strong influence of physician satisfaction on adherence, echoing the literature emphasizing the role of trust and communication in chronic disease management. Patients who felt supported by their doctors were more likely to adhere to their treatment, underscoring the need for patient-centered communication strategies. This aligns with another Saudi study conducted by Khan et al. in Al-Hasa district [21]. This positive influence could be attributed to successful patient–physician communication, characterized by support, simplified essential information, and active listening, which subsequently fosters a regular follow-up routine.

Furthermore, beliefs and perceptions significantly impacted adherence. Patients who rejected misconceptions, such as viewing diabetes medications as addictive or only necessary when blood sugar is high, were consistently more adherent. This highlights the importance of targeted education to dispel myths and reinforce the chronic nature of diabetes.

This study could shed light on the importance of improving patient education regarding the management of type 2 diabetes. Primary healthcare providers can target the factors leading to non-adherent behavior to avoid patient complications and reduce hospitalization rates and mortality.

### Future Perspectives

Although our findings add to the growing body of literature on diabetes care in the Middle East, future research should explore additional cultural factors prevalent in this region, such as the dynamics of family involvement in patient care and healthcare expectations, as these may profoundly influence medication adherence behaviors and provide further insights.

## 5. Limitations

Our study has several limitations, including its cross-sectional nature, which could lead to recall and social desirability biases, thereby limiting the establishment of causal relationships between the variables. Not all factors were assessed, such as the availability and cost of medications. Additionally, this study employed a cross-sectional design with convenience sampling techniques, which may limit the generalizability of the findings, as cultural beliefs can vary significantly from one population to another. Future studies should preferably employ a prospective longitudinal design with more confounding variables for more precise findings.

## 6. Conclusions

In conclusion, medication adherence among diabetic patients in Madinah was generally moderate. According to the MMAS-4, 50% of participants exhibited medium adherence, with 28.8% classified as high adherers and 21.1% classified as low adherers. According to the GMAS, 36.6% demonstrated high adherence, and 34.4% demonstrated good adherence. Additionally, this study identified possible influencing factors, including patient characteristics such as age, the presence of comorbidities, educational level, patient satisfaction with their physicians, and health self-awareness. Healthcare providers and policymakers should collaborate to implement health educational campaigns to raise awareness of diabetes medication adherence and self-management to achieve better clinical outcomes in diabetic patients.

## Figures and Tables

**Table 1:** Participants’ demographic characteristics (N = 424).

Factor	Category	Number	Percentage
Age (years)	9–39	125	29.5
	40–59	204	48.1
	≥60	95	22.4
Gender	Male	158	37.3
	Female	266	62.7
BMI (kg/m^2^)	Underweight/normal < 25	149	35.1
	Overweight 25–29.9	145	34.2
	Obese ≥ 30	130	30.7
Nationality	Saudi	401	94.6
	Non-Saudi	23	5.4
Residence	Urban	384	90.6
	Rural	40	9.4
Smoker	Yes	48	11.3
	No	376	88.7
Level of education	Illiterate	35	8.3
	Elementary	40	9.4
	Intermediate/secondary	98	23.1
	University	251	59.2
Employment status	Unemployed	207	48.8
	Employed	144	34.0
	Retired	73	17.2
Marital status	Single	97	22.9
	Married	271	63.9
	Divorced	17	4.0
	Widowed	39	9.2
Live alone	Yes	49	11.6
	No	375	88.4
Someone is helping with your diabetes care	Yes	268	63.2
	No	156	36.8
Monthly family income (SAR)	<5000	130	30.7
	5000–10,000	125	29.5
	>10,000	169	39.9
Health insurance coverage	Yes	103	24.3
	No	321	75.7
Comorbidities	Yes	151	35.6
	No	273	64.4
If yes, type of comorbidity	Hypertension	104	24.5
	Hypothyroidism	31	7.3
	Cardiovascular disease	20	4.7
	Asthma	7	1.7
	Other *	20	4.7

* Other: high cholesterol, systemic lupus erythematosus, epilepsy, sickle cell anemia, renal failure, hepatitis C virus, cancer, osteoporosis, psoriasis, rheumatoid arthritis, celiac disease, liver cirrhosis, Behcet disease, colon cancer, benign prostatic hyperplasia, cataract.

**Table 2:** Participants’ clinical and diabetes medication profiles (N = 424).

Factor	Category	Number	Percentage
Total number of medications	1–2	184	43.4
	3–4	124	29.2
	≥5	116	27.4
Diabetes type	Type 1	134	31.6
	Type 2	290	68.4
Diabetes duration (years)	<5	152	35.8
	5–10	87	20.5
	>10	185	43.6
Family history of diabetes	Yes	310	73.1
	No	114	26.9
Diabetic complications	Yes	199	46.9
	No	225	53.1
If yes, type of complications	Retinopathy	122	28.8
	Neuropathy	75	17.7
	Oral diseases	46	10.8
	Skin diseases	45	10.6
	Nephropathy	30	7.1
	Cardiovascular complications	22	5.2
	Sexual problems	22	5.2
	Diabetic foot	18	4.2
	Other **	4	0.8
HbA1c %	Normal: <5.7%	14	3.3
	Prediabetes: 5.7–6.4%	54	12.7
	Diabetes: ≥6.5%	354	84.0
HbA1c control (N = 423)	Controlled < 6.5%	184	43.5
	Uncontrolled ≥ 6.5%	239	56.5
Type of treatment	Insulin only	127	30.0
	Oral only	213	50.2
	Both	84	19.8
Number of diabetes medications prescribed	1	155	36.6
	2	156	36.8
	3	66	15.6
	4 or more	47	11.1
Do you take any herbal medication?	Yes	88	20.8
	No	336	79.2
If yes, what is it?	Cinnamon	36	8.5
	Fenugreek	18	4.2
	Coriander	13	3.1
	Ginger	12	2.8
	Moringa	6	1.4
	Cumin	5	1.2
	Chamomile	5	1.2
	Anise	4	0.9
	Rosemary	4	0.9
	Olive leaves	4	0.9
	Other ***	32	7.5
Have you ever been instructed on diabetes care?	Yes	326	76.9
	No	98	23.1
Have you ever received education on diabetes?	Yes	310	73.1
	No	114	26.9
Do you exercise regularly?	Yes	135	31.8
	No	289	68.2
Do you see your doctor regularly?	Yes	265	62.5
	No	159	37.5

** Other: bone pain, general fatigue, osteoarthritis, stroke. *** Other: green tea, fennel, hibiscus, parsley, Artemisia, arugula, clove, Fagonia, felty germander, fig leaves, garlic, Gymnema sylvestre, Indian costus, lemon, linseed, marjoram, myrrh, okra soak, turmeric, mixed herbs. HbA1c control based on n = 423 participants with available lab values; all other variables based on full sample (N = 424).

**Table 3:** Participants’ beliefs towards diabetes mellitus (N = 424).

Factor	Category	Number	Percentage
Are you satisfied with your doctor?	Yes	359	84.7
	No	65	15.3
Do you believe you have diabetes only when your blood sugar is high?	Agree	191	45.0
	Disagree	233	55.0
The consequences of diabetes are low	Agree	48	11.3
	Disagree	376	88.7
Symptoms of diabetes are minimal	Agree	74	17.5
	Disagree	350	82.5
Low control over diabetes	Agree	238	56.1
	Disagree	186	43.9
Don’t need diabetes medicines when sugar is normal	Agree	183	43.2
	Disagree	241	56.8
Worried about the side effects of medicines	Agree	291	68.6
	Disagree	133	31.4
Worried about addiction to medicines	Agree	228	53.8
	Disagree	196	46.2
Do you find it difficult to take your diabetes medications?	Agree	147	34.7
	Disagree	277	65.3
Little confidence in the ability to control diabetes	Agree	200	47.2
	Disagree	224	52.8
Have significant depressive symptoms	Agree	152	35.8
	Disagree	272	64.2
Diabetes significantly interferes with social life	Agree	230	54.2
	Disagree	194	45.8

**Table 4:** Impact of diabetic patients’ sociodemographic and clinical characteristics on medication adherence (N = 242).

Factor	Category	GMAS Median (IQR)	*p*-Value	MMAS Median (IQR)	*p*-Value
Age (years)	9–39	24 (12)	**<0.001**	**2 (2)**	**0.004**
	40–59	28 (9)		**2 (2)**	
	≥60	**29 (10)**		**3 (2)**	
Gender	Male	26 (10)	0.604	2 (2)	0.917
	Female	28 (10)		2 (2)	
BMI (kg/m^2^)	Underweight/normal < 25	25 (9)	**0.023**	2 (2)	0.715
	Overweight 25–29.9	**28 (9)**		2 (2)	
	Obese ≥ 30	27 (10)		2 (2)	
Nationality	Saudi	**28 (10)**	**0.015**	2 (2)	**0.015**
	Non-Saudi	24 (8)		**2 (3)**	
Residence	Urban	27 (10)	0.491	2 (2)	0.298
	Rural	28 (12)		3 (3)	
Smoker	Yes	22.5 (10)	0.072	2 (2)	0.291
	No	28 (10)		2 (2)	
Level of education	Illiterate/lower degree	28 (9)	0.103	2 (2)	**0.027**
	University	26 (10)		**2 (2)**	
Employment status	Unemployed	27 (9)	0.364	2 (2)	0.570
	Employed/retired	27 (10)		2 (2)	
Marital status	Single/divorced/widowed	26 (11)	0.092	2 (2)	0.338
	Married	28 (9)		2 (2)	
Live alone	Yes	28 (9)	0.298	3 (2)	0.348
	No	27 (10)		2 (2)	
Someone is helping with your diabetes care	Yes	27 (10)	0.836	2 (2)	0.380
	No	27.5 (9)		2 (2)	
Monthly family income (SAR)	<5000	26 (10)	0.797	2 (2)	0.998
	5000–10,000	27 (10)		2 (2)	
	>10,000	28 (10)		2 (2)	
Health insurance coverage	Yes	27 (13)	0.347	2 (2)	0.153
	No	27 (9)		2 (2)	
Comorbidities	Yes	**29 (10)**	**0.005**	2 (3)	0.099
	No	26 (9)		2 (2)	
Total number of medications	1–2	26 (9)	**<0.001**	**2 (2)**	**<0.001**
	3–4	25 (11)		**2 (2)**	
	≥5	**30 (7)**		**3 (2)**	
Diabetes type	Type 1	26 (11)	**0.024**	2 (2)	0.916
	Type 2	**28 (9)**		2 (2)	
Diabetes duration (years)	<5	25 (12)	**0.002**	**2 (2)**	**0.001**
	5–10	28 (10)		2 (1)	
	>10	**28 (9)**		2 (2)	
Family history of diabetes	Yes	27 (9)	0.953	2 (2)	0.281
	No	28 (10)		2 (1)	
Diabetic complications	Yes	26 (9)	0.268	2 (2)	0.225
	No	28 (10)		2 (2)	
HbA1c %	Normal: <5.7%	30 (6)	0.370	3 (2)	0.060
	Prediabetes: 5.7–6.4%	27.5 (11)		2 (3)	
	Diabetes: ≥6.5%	27 (10)		2 (2)	
HbA1c control (n = 423)	Controlled < 6.5%	28 (11)	0.678	2 (3)	0.365
	Uncontrolled ≥ 6.5%	27 (9)		2 (2)	
Type of treatment	Insulin only	26 (11)	0.367	2 (2)	0.616
	Oral only	28 (9)		2 (2)	
	Both	27 (10)		2 (3)	
Number of diabetes medications prescribed	1	24 (10)	**0.008**	**2 (2)**	**0.043**
	2	**28 (9)**		2 (2)	
	3 or more	27 (9)		**2 (2)**	
Do you take any herbal medication?	Yes	26 (8)	**0.025**	**2 (2)**	**0.007**
	No	**28 (10)**		2 (2)	
Have you ever been instructed on diabetes care?	Yes	27 (10)	0.740	2 (2)	0.942
	No	27 (9)		2 (2)	
Have you ever received education on diabetes?	Yes	27 (9)	0.528	2 (2)	0.517
	No	27 (12)		2 (3)	
Do you exercise regularly?	Yes	27 (11)	0.523	2 (1)	0.253
	No	27 (9)		2 (2)	
Do you see your doctor regularly?	Yes	**28 (9)**	**<0.001**	2 (2)	**<0.001**
	No	24 (10)		**2 (2)**	

**Table 5:** Impact of diabetic patients’ beliefs on medication adherence (N = 242).

Factor	Category	GMAS	*p*-Value	MMAS	*p*-Value
Are you satisfied with your doctor?	Yes	**28 (9)**	**0.008**	2 (2)	0.215
	No	23 (11)		2 (2)	
Do you believe you have diabetes only when your blood sugar is high?	Agree	25 (11)	**0.022**	2 (2)	0.230
	Disagree	**28 (9)**		2 (1)	
The consequences of diabetes are low	Agree	23 (18)	**0.010**	2 (2)	0.168
	Disagree	**28 (9)**		2 (2)	
Symptoms of diabetes are minimal	Agree	26.5 (16)	0.191	2 (2)	0.558
	Disagree	27 (9)		2 (2)	
Low control over diabetes	Agree	25 (10)	**<0.001**	**2 (2)**	**<0.001**
	Disagree	**29 (8)**		**3 (2)**	
Don’t need diabetes medicines when sugar is normal	Agree	24 (11)	**<0.001**	**2 (2)**	**<0.001**
	Disagree	**29 (10)**		**3 (2)**	
Worried about the side effects of medicines	Agree	26 (9)	**<0.001**	**2 (2)**	**<0.001**
	Disagree	**30 (9)**		**3 (2)**	
Worried about addiction to medicines	Agree	26 (11)	**<0.001**	2 (2)	**<0.001**
	Disagree	**29 (9)**		**3 (1)**	
Do you find it difficult to take your diabetes medications?	Agree	22 (10)	**<0.001**	**2 (3)**	**<0.001**
	Disagree	**29 (7)**		**3 (1)**	
Little confidence in the ability to control diabetes	Agree	23 (10)	**<0.001**	**2 (2)**	**<0.001**
	Disagree	**29 (8)**		**3 (2)**	
Have significant depressive symptoms	Agree	22.5 (12)	**<0.001**	**2 (2)**	**<0.001**
	Disagree	**29 (8)**		**3 (1)**	
Diabetes significantly interferes with social life	Agree	24 (10)	**<0.001**	**2 (2)**	**<0.001**
	Disagree	**29.5 (8)**		**3 (2)**	

## Data Availability

The original contributions presented in this study are included in the article. Further inquiries can be directed to the corresponding author.
