# Correction: Albadrani et al. Medication Adherence Among Diabetic Patients in Madinah, Saudi Arabia: Interplay of Cultural Beliefs, Socioeconomic Status, and Clinical Determinants. *J. Clin. Med.* 2025, *14*, 6717

**DOI:** 10.3390/jcm15197599

**Published:** 2026-09-30

**Authors:** Muayad Albadrani, Asrar Alharbi, Shahad Aljohani, Reenad Al Harbi, Taif Alluhaybi, Esraa Alammash, Afrah Aljabri, Naweed SyedKhaleel Alzaman

**Affiliations:** 1 Department of Family and Community Medicine and Medical Education, College of Medicine, Taibah University, Madinah 42353, Saudi Arabia; 2 Health and Life Research Center, Taibah University, Madinah 42353, Saudi Arabia; 3 College of Medicine, Taibah University, Madinah 42353, Saudi Arabia; 4 Department of Medicine, College of Medicine, Taibah University, Madinah 42353, Saudi Arabia

**Text Correction**

There was an error in the original publication [1]. The original article described the MMAS-4 scoring in a reversed manner (high adherence = 0, low = 4). To align with the validated MMAS-4 scale (High Adherence: Score = 4, Medium Adherence: Score = 2–3, and Low Adherence: Score = 0–1), the scoring method was corrected. This correction affects only the scoring description and does not alter the meaning, interpretation, or statistical significance of the study’s results or conclusions.

A correction has been made to Section 2. Methods, 2.3. Data Collection Method and Tools, Paragraph Number 1 as follows:

The Morisky Medication Adherence Scale-4 (MMAS-4), specifically adapted for diabetes medication adherence, was utilized. This scale comprises four items that assess various aspects of medication-taking behavior, including forgetting, carelessness, stopping medication when feeling better, and stopping medication when feeling worse. The term “modified” refers to its specific application and interpretation in the context of diabetes management, drawing on established factors relevant to adherence in this patient population, as identified in previous research. Participants were divided into two groups based on their adherence scores: 4 (high adherence) and 3 to 0 (medium or low adherence). 

In addition, Section 2. Methods, 2.4. Statistical Analysis, Paragraph Number 2 was updated to reflect the same scoring scale as follows:

The Morisky Medication Adherence Scale (MMAS-4) was utilized to assess the level of adherence to diabetes medication. Respondents who answer “no”, indicating the patient did not exhibit the non-adherent behavior, receive a score of 1, while those who answer “yes” receive a score of 0. Data have been coded using validated cutoffs (High = 4, Medium = 2–3, Low = 0–1). 

For the title of Figure 3, the categories were updated to: high (4), medium (2–3), or low (0–1).

In addition, some median (IQR) MMAS-4 adherence scores reported in Tables 4 and 5 were updated to align with the corrected scoring system, where scores of 1 were reclassified as 3 and vice versa.

The authors state that the scientific conclusions are unaffected. This correction was approved by the Academic Editor. The original publication has also been updated.
